# Complete Chloroplast Genome Sequence of *Malus hupehensis*: Genome Structure, Comparative Analysis, and Phylogenetic Relationships

**DOI:** 10.3390/molecules23112917

**Published:** 2018-11-08

**Authors:** Xin Zhang, Chunxiao Rong, Ling Qin, Chuanyuan Mo, Lu Fan, Jie Yan, Manrang Zhang

**Affiliations:** College of Horticulture, Northwest A&F University, Yangling 712100, Shaanxi, China; zzspch@163.com (X.Z.); rongchunxiao501@163.com (C.R.); 18392101060@163.com (L.Q.); mochuanyuan@163.com (C.M.); 18829716820@163.com (L.F.); 18792979391@163.com (J.Y.)

**Keywords:** *Malus hupehensis*, chloroplast genome, comparative analysis, phylogenetic analysis

## Abstract

*Malus hupehensis* belongs to the *Malus* genus (Rosaceae) and is an indigenous wild crabapple of China. This species has received more and more attention, due to its important medicinal, and excellent ornamental and economical, values. In this study, the whole chloroplast (cp) genome of *Malus hupehensis*, using a Hiseq X Ten sequencing platform, is reported. The *M. hupehensis* cp genome is 160,065 bp in size, containing a large single copy region (LSC) of 88,166 bp and a small single copy region (SSC) of 19,193 bp, separated by a pair of inverted repeats (IRs) of 26,353 bp. It contains 112 genes, including 78 protein-coding genes (PCGs), 30 transfer RNA genes (tRNAs), and four ribosomal RNA genes (rRNAs). The overall nucleotide composition is 36.6% CG. A total of 96 simple sequence repeats (SSRs) were identified, most of them were found to be mononucleotide repeats composed of A/T. In addition, a total of 49 long repeats were identified, including 24 forward repeats, 21 palindromic repeats, and four reverse repeats. Comparisons of the IR boundaries of nine *Malus* complete chloroplast genomes presented slight variations at IR/SC boundaries regions. A phylogenetic analysis, based on 26 chloroplast genomes using the maximum likelihood (ML) method, indicates that *M. hupehensis* clustered closer ties with *M. baccata*, *M. micromalus*, and *M. prunifolia* than with *M. tschonoskii*. The availability of the complete chloroplast genome using genomics methods is reported here and provides reliable genetic information for future exploration on the taxonomy and phylogenetic evolution of the *Malus* and related species.

## 1. Introduction

Chloroplasts are important organelles involved in photosynthesis, supplying the indispensable energy for plant growth and development. The chloroplast genome typically has a quadripartite organization, with a LSC region, a SSC region and two identical copies of IR regions [1]. In angiosperms, the most complete cp genome sizes range from 120 to 160 kb [2]. Apart from its quadripartite structure, about 100–130 genes were included in chloroplast genome, and therefore the performance in their composition and arrangement are very conservative [3]. The chloroplast DNA shows maternal inheritance in most plant species, less recombination and has a slow rate of evolution, which is substantially different from the nuclear genome [4] that has been widely applied in evolutionary relationships at the taxonomic level in plants. The cp DNA genome Sequencing can support knowledge for researching the molecular evolutionary, RNA Editing, population genetics, and transplastomic studies [5,6,7,8,9]. With the development of next-generation sequencing technologies, provides a cost-effective means and efficiently get complete chloroplast genome information, which can contribute to the resolution of species relationships. Moreover, the comparative analysis of chloroplast genomes can contribute to a theoretical basis for a phylogenetic status study [10,11].

*Malus* Miller is an economically important genus of about 62 species (http://www.theplantlist.org/1.1/browse/A/Rosaceae/Malus/). The genus *Malus* Miller (Rosaceae) are widely found in the Northern Hemisphere temperate zone [12]. About 30 to 35 species of the *Malus* genus are widely distributed in China [13]. Species of the *Malus* genus are well known for their leaves, flower and fruit, which have great value in the medicinal, agricultural product, and food handling industries [14,15]. The *Malus* fruit and related products, such as cider, vinegar or juice, are well received by consumers. Numerous studies have shown that compounds in *Malus* plants have a medicinal tonic function and therapeutic role [16,17]. Additionally, the plants of the *Malus* genus are used as materials that can potentially be used for the production of nutraceuticals and cosmeceuticals. The *Malus* species have an excellent horticultural trait that is used as an experimental research plant material, which is of great value to researchers. Previous studies have used microsatellite markers to assess a broad range of genetic diversity resources in *Malus* germplasm collections [18]. Additionally, in morphological and biochemical diversity analyses from the parts of *Malus* species, phylogenetic relationships have been conducted, however, the number of them is limited [19,20,21]. However, the taxonomy of the *Malus* genus is complex and unclear, and in light of new genomic resources, in need of revision [22]. Therefore, the *Malus* species complete chloroplast genome databases can make the contribution of a useful resource for researchers in identifying species, plant genetic improvements, biotic and abiotic resistance evaluations, and research on cell physiology and biochemistry.

*Malus hupehensis*, an indigenous wild crabapple cultivar of the *Malus* genus, grows naturally in the forests of slopes or valley thickets at an elevation of 50–2900 m and is widely distributed throughout China [12]. As an important traditional Chinese medicinal material, it is used to treat ailments related to the spleen stomach, and constipation [23,24]. The extracts of *M. hupehensis* possess abundant bioactive compounds, such as polyphenols, flavonoids and chalcon, which have the pharmacological action of potent anti-oxidative, anti-microbial, anti-inflammation and anti-fatigue properties [25,26,27]. Among these beneficial bioactive compounds from the *M. hupehensis*, polyphenols can significantly lower plasma glucose levels [28], flavonoids can protect doxorubicin-induced cell apoptosis and inhibit the occurrence of liver fibrosis [28,29]. Moreover, the young leaves of this plant are used for a tea drink in China due to being rich in a variety of essential trace elements of the human body, which have healthy activities and are very popular with people [30]. It has charming flowers in the spring, attractive foliage in the summer, beautiful fruit in the autumn, and is a steadfast component of the landscape industry that is widely cultivated. Furthermore, *M. hupehensis* is also a common apple rootstock, with apomixis traits, strong disease resistance, strong resistance to stress, strong grafting affinity with the main variety and a certain dwarfing effect [31].

Here, we sequenced the *M. hupehensis* cp genome applying Illumina sequencing technology and analyzed the genome features, and this was the first comprehensive complete cp genome analysis of *M. hupehensis*, combined with the whole cp genome sequences of eight other *Malus* species, previously published. Furthermore, we also used 26 complete cp genome sequences from GenBank to construct the phylogenetic relationships and infer the phylogenetic position of *M. hupehensis*. Our data will provide valuable information for further studies. Meanwhile, the data can contribute to the exploration and utilization of *Malus* plants.

## 2. Results and Discussions

### 2.1. Chloroplast Genome Features of M. hupehensis

**Figure 1:**
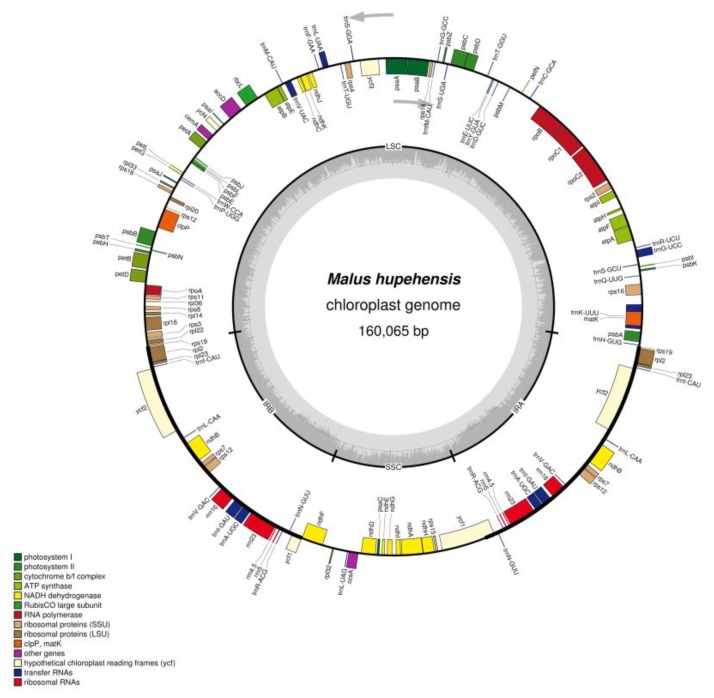
Gene map of the *M. hupehensis* chloroplast genome. Genes shown outside the outer circle are transcribed clockwise and those inside are transcribed counterclockwise. The colored bars indicate different functional groups. The dark gray inner circle corresponds to the GC content, the light-gray to the AT content.

We acquired approximately 7.3 Gb reads for *M. hupehensis* were through the Illumina HiSeq X Ten system (Illumina, San Diego, CA, USA). The complete cp genome sequence of *M. hupehensis* had been deposited into GenBank (No. MK020147). *M. hupehensis* cp genome has a quadripartite architecture, and has 160,065 nucleotides, which are geared to the size of a landplant cp genome [32], consisting of a pair of IRs (26,353 bp), a SSC region (19,193 bp) and a LSC region (88,166 bp), which is similar to other *Malus* complete chloroplast genomes (Table 1 and Figure 1). The GC content of the LSC (34.2%) and SSC regions (30.4%) was lower than that in IR regions (42.7%). The relatively high GC content of the IR regions was mostly attributable to the four rRNAs and tRNAs [33,34]. Additionally, the GC percentage in *M. hupehensis* complete chloroplast genome was 36.6%, which nearly the same as in the other eight *Malus* complete chloroplast genomes (Table 1).

The studied chloroplast genomes of green plants usually comprise 110–130 genes, of which ~80 are PCGs, ~30 are tRNAs and four are rRNAs [35]. In the *M. hupehensis* chloroplast genome, 131 functional genes were identified, the positions of those genes are shown in Figure 1, which has 112 unique genes (Table 2), including 78 PCGs, 30 tRNAs, and four rRNAs. Among of those, six protein-coding genes (*ndhB*, *rpl2*, *rpl23*, *rps7*, *rps12* and *ycf2*), seven tRNA genes (*trnA-UGC*, *trnL-CAA*, *trnI-GAU*, *trnI-CAU*, *trnN-GUU*, *trnV-GAC*, *trnR-ACG*), and four rRNA genes (*4.5S*, *5S*, *16S*, *23S*) are located in IR regions, which were totally duplicated. Moreover, a total of 62 PCGs and 22 tRNA genes were located in the LSC region, also, there were 11 PCGs and one tRNA gene located in the SSC region.

Among these annotated genes, a total of 15 genes (*atpF*, *ndhA*, *ndhB*, *petB*, *petD*, *rpl16*, *rpl2*, *rpoC1*, *rps16*, *trnA-UGC*, *trnG-GCC*, *trnI-GAU*, *trnK-UUU*, *trnL-UAA*, *trnV-UAC*) contained one intron, and three genes (*clpP*, *rps12*, and *ycf3*) contained two introns (Table 3). The *clpP* gene is essential for chloroplast development, which encodes ATP-dependent protease proteolytic subunit [36]. The past study have reported that the *clpP* splicing efficiency was increased under drought stress [37]. The *clpP* of *M. hupehensis* may be useful for further studies of this plant’s response to abiotic stresses in apple rootstock. A trans-spliced gene, with a 5′ exon situated in the LSC region and the duplicated 3′ end in the IR region, which is conserved in most other land plants [38], is found in *rps12*. The *trnL-UAA* was provided with the smallest intron (514 bp), whereas the intron of *trnK-UUU* possesses the largest intron (2497 bp), the *matK* gene is contained in it. Meanwhile, the *matK* gene is widely used as a molecular marker to research the phylogenetic relationships in other angiosperms [39,40,41,42,43]. Additionally, in previous studies, the *matK* region of *Malus* cp genome had been analyzed to contribute to the identification of potential germplasm donors for the cultivated *Malus* species [22].

**Figure 2:**
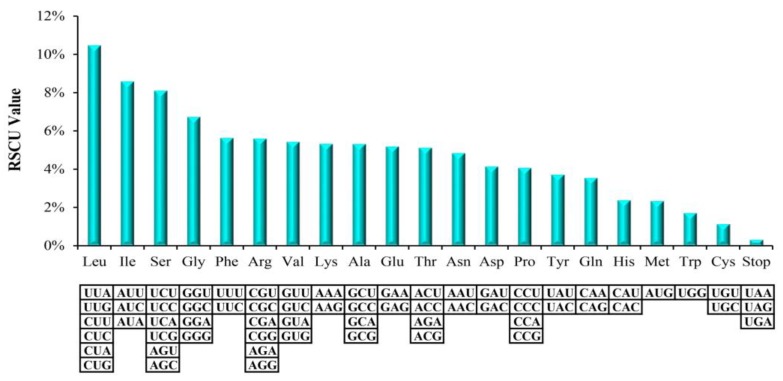
Codon content of 20 amino acid and the stop codon of 84 coding genes of the *M. hupehensis* cp genome.

Relative synonymous codon usage (RSCU) values as an availability source, which can make for the phylogenetic relationship studies [44]. The synonymous codons in angiosperms genomes possess usage frequencies differently, that is, a codon usage bias, which is a significant evolutionary character within genome that can provide essential information for studying organism evolution [45]. In the *M. hupehensis* chloroplast genome, the all PCGs included 78,564 bp that encoded codons numbers are 26,188. Among all these codons, there are up to 2747 (10.49%) codons encoded leucine. However, only a small amount of codons (300, 1.15%) encoded cysteine (Table S1, Figure 2). Of course, the used amino acids of leucine and cysteine were the most and the least frequently in the *M. hupehensis* cp genome, respectively. The use of the starting codon methionine AUG and tryptophan UGG had no bias (RSCU = 1). Moreover, 31 codons ending with A or U, which contained 29 preferred synonymous codons (RSCU > 1.0), the rest are *trnL-UAG* (RSCU = 0.78), *trnI-CAU* (RSCU = 0.95) and a stop codon (UAG) (RSCU = 0.54) (Table S1).

### 2.2. SSR and Long-Repeat Analysis

**Figure 3:**
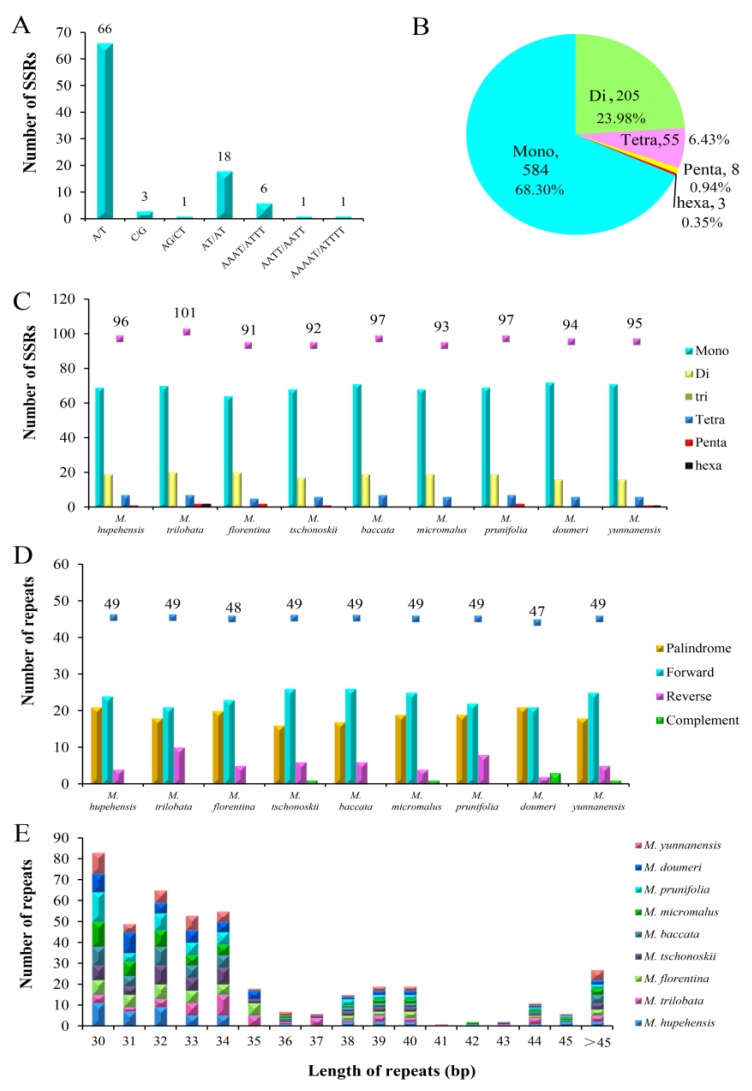
Repeat analyses. (**A**) Repeat unit and amounts of SSR in the *M. hupehensis* cp genome. (**B**) Presence of different SSR types in all of the SSRs of nine *Malus* chloroplast genomes. (**C**) SSRs in the nine *Malus* cp genomes. (**D**) Repeated sequences in the nine *Malus* cp genomes. (**E**) Repeat frequency of four types by length in the nine *Malus* chloroplast genomes.

Simple sequence repeats, with high rate of mutation and diversity copy number, as shown by molecular markers for genetic diversity and evolutionary reseaches [46,47]. In a previous study, SSR markers were used to identify the germplasm and genetic relationship of *M. hupehensis* [31]. With MISA analysis, a total of 96 SSRs were identified, and there were 69, 19, 7, and 1, mononucleotide, dinucleotide, tetranucleotide, and pentanucleotide repeats, respectively (Figure 3A). These SSRs are very conducive to the Rosaceae complete chloroplast genomes A/T abundance [48,49,50]. In addition, the A/T mononucleotide repeats 69 (71.88%) were the most common. This result is in agreement with previous studies showing that the most abundant SSR pattern was generally composed of mononucleotides (A/T) [48]. Mononucleotides in all of the SSRs of nine *Malus* chloroplast genomes with the highest proportion reached 68.30%, followed by the dinucleotides (23.98%), tetranucleotides (6.43%), pentanucleotides (0.94%) and, finally, the hexanucleotide (0.35%) (Figure 3B). There were no trinucleotide repeats observed in all 9 *Malus* species. In all, 856 repeats were detected in the nine *Malus* species. The numbers of the SSR repeats were 96, 101, 91, 92, 97, 93, 97, 94, and 95 in *M. hupehensis*, *M. trilobata*, *M. florentina*, *M. tschonoskii*, *M. baccata*, *M. micromalus*, *M. prunifolia*, *M. doumeri*, and *M. yunnanensis*, respectively (Figure 3C). The results of these studies will allow chloroplast SSR markers to be used in the study of the genetic diversity in *M. hupehensis*, which can be valuable for comparing phylogenetic relationships and inferring the population genetic structure among related *Malus* species.

In total, 49 repeats were identified of chloroplast genome of *M. hupehensis*, including 24 forward repeats, 21 palindromic repeats, and four reverse repeats. This result agrees with the eight other *Malus* complete cp genomes, which vary in numbers, from 47 to 49. Of all nine *Malus* species, forward is the most abundant repeat type, palindrome and reverset are close behind; complements were detected in *M. tschonoskii*, *M. micromalus*, *M. doumeri*, and *M. yunnanensis*, and numbers of them were 1, 1, 3, and 1, respectively (Figure 3D). Most of these repeats were mainly fall within 30 bp to 40 bp. Furthermore, the maximum and minimum length are 69 and 30, respectively, and most of them are within this range for each species (Figure 3E). In *M. hupehensis* cp genome, we found that most repeats are situated in intergenic sequences (Table S2), which was in keeping with the other research results [51].

### 2.3. IR Contraction and Expansion

**Figure 4:**
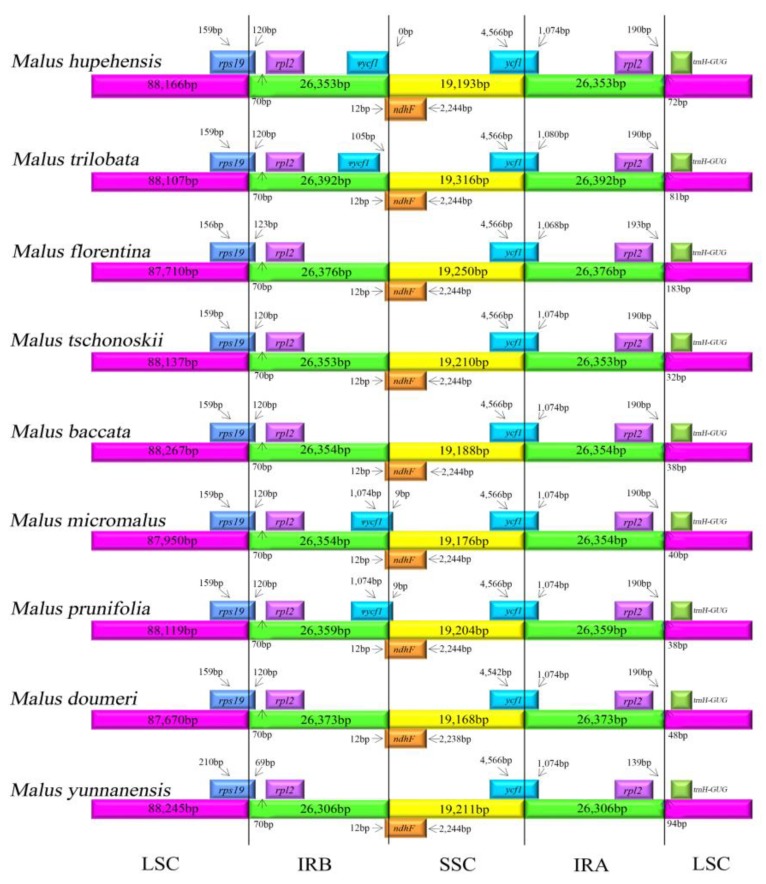
Comparison of the border positions of LSC, SSC, and IR regions among the nine *Malus* chloroplast genomes.

The IR boundary expansion and contraction is deemed to an evolutionary event and has been shown to be the primary mechanism of the size variation of chloroplast genomes in higher plants [52,53]. In this study, the junctions between the IR and LSC/SSC regions among the nine *Malus* chloroplast genomes were compared (Figure 4). The chloroplast genomes are highly conserved, although there are also slight length discrepancies between the nine chloroplast genomes. Some expansion and contraction was presented in *M. hupehensis* IR region lengths and other *Malus* species, with the IR regions ranging from 26,306 bp in *M. yunnanensis* to 26,392 bp in *M. trilobata* (Table 1). For the LSC/IR borders, the gene *rps19* in the LSC of all complete chloroplast genomes extended from 69–120 bp into the IRb region. In *M. hupehensis*, *M. trilobata*, *M. micromalus*, and *M. prunifolia* complete chloroplast genomes, the *ycf1* in the IRb regions was a long way from the IRb/SSC junction, 105 bp from the junction in *M. trilobata* and 0 bp from *M. hupehensis*, whereas it shifted by an identical distance (9 bp) from LSC to IRb at the LSC/IRb border in *M. micromalus* and *M. prunifolia*. Furthermore, the photosynthetic gene, *ndhF*, extended into the LSC region by 12 bp in all species. The position of *ycf1* in the IRa regions varied from 1068 to 1080 bp. Similarly, the IRa/LSC border is located between the *rpl2* and *trnH* genes, and the *trnH* gene is located in the LSC region, 72, 81, 183, 32, 38, 40, 38, 48, and 94 bp away from the IRa/LSC border in the nine *Malus* cp genomes (*M. hupehensis*, *M. trilobata*, *M. florentina*, *M. tschonoskii*, *M. baccata*, *M. micromalus*, *M. prunifolia*, *M. doumeri* and *M. yunnanensis*), respectively. The *trnH* gene in the LSC regions was 183 bp from the IRb/SSC border of *M. florentina*, which is much further than in other species. In general, among these nine *Malus* species cp genome, there is a slight difference in IR boundary regions.

### 2.4. Comparative Chloroplast Genomic Analysis

**Figure 5:**
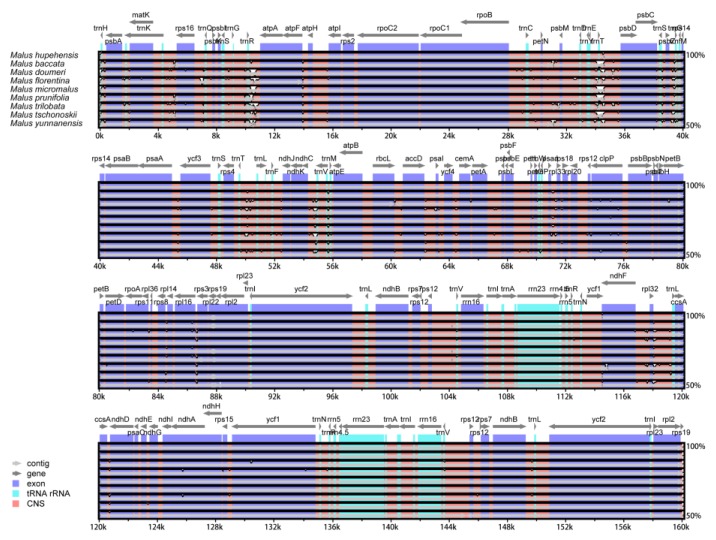
Comparison of nine cp genomes using mVISTA. The chloroplast genome of *M. hupehensis* as a reference. The grey arrows and thick black lines above the alignment indicate the position and direction of each gene. The y-axis represents the percentage identity (shown: 50–100%).

The comparative analysis of chloroplast genome can provide knowledge of complex evolutionary relationships [54]. In the present study, eight *Malus* chloroplast genomes, and *M. hupehensis* chloroplast genome were compared (Figure 5). The nine *Malus* cp genomes length between the confines of 159,584 to 160,207 bp. The chloroplast genome of *M. trilobata* has the largest size, whereas the chloroplast genome size of *M. doumeri* is the smallest. All nine *Malus* complete chloroplast genomes indicated that the length of IR regions ranges from 26,306–26,392 bp, that of the LSC regions ranges from 87,670–88,267 bp, and that of the SSC regions ranges from 19,168–19,316 bp, and all species showed a similar size in the LSC, SSC, and IR regions (Table 1). The complete chloroplast genome of *M. hupehensis* was compared with eight other genomes using the mVISTA program with a Shuffle-LAGAN model to investigate the level of sequence divergence, the alignment of which showed that the nine chloroplast genomes were conserved, with a high degree of synteny and gene order (Figure 4). However, some divergence was found within the intergenic spacers and introns of these nine chloroplast genomes, including *trnH-psbA*, *trnK-rps16*, *rps16-trnQ*, *trnS-trnG*, *trnR-atpA*, *petN-psbM*, *trnE-trnT*, *trnT-psbD*, *trnS-psbZ*, *psbZ-trnG*, *psaA-ycf3*, *trnT-trnL*, *ndhC-trnV*, *rps8-rpl14*, *rpl16-rps3*, *ndhF-rpl32*, *rps32-trnL*, *ccsA-ndhD*, as well as *trnV*, *ndhA*, and *clpP* introns. Additionally, the results of this study shown that the coding regions were more highly conserved than the non-coding regions, and IRs had a lower sequence divergence than the LSC and SSC regions, which is identical with other angiosperms [55]. The dissimilar coding regions of the nine cp genomes were *matK*, *rpoA*, *ndhF*, and *ycf1*, which are barcodes for land plants that have been indicated in past studies [56,57,58,59]. The possibility of further studying the trend of these regions used as molecular markers will allow for a deeper investigation of the phylogenetic development of the *Malus*.

### 2.5. Phylogenetic Analysis

**Figure 6:**
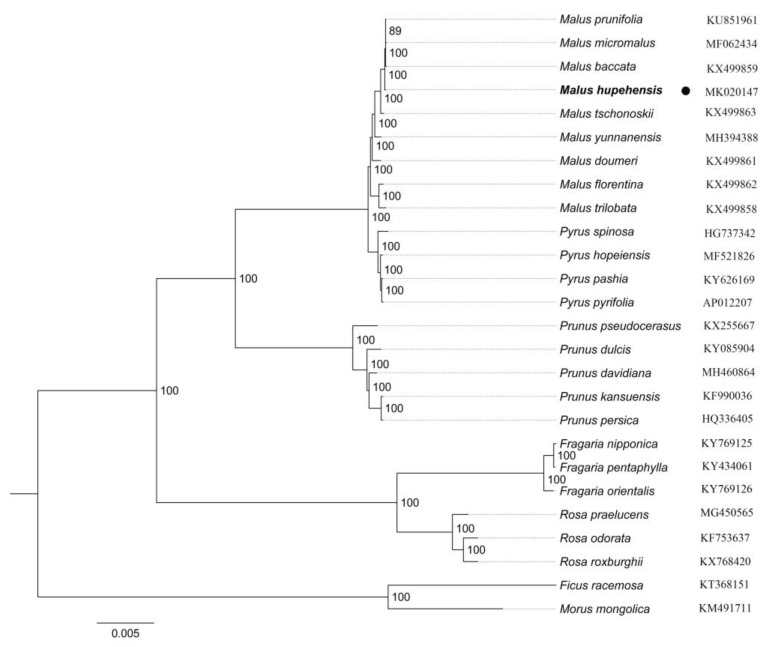
A maximum likelihood (ML) phylogenetic tree based on 26 species chloroplast genomes was constructed. *Ficus racemosa* and *Morus mongolica* (Moraceae) were used as the outgroup.

Past research has shown that the chloroplast genome of terrestrial plants have been as a valuable source among related species, which is applied in phylogenetic studies [60,61]. In this paper, we completed an alignment of all chloroplast genomes of 26 species, which included nine *Malus* species, four *Pyrus* species, five *Prunus* species, three *Fragaria* species and three *Rosa* species, and two Moraceae species. As shown in the phylogenetic tree, *Malus* was closely related to *Pyrus* than with *Prunus*. *Malus* and *Pyrus* are included in the Maleae, and Prunoideae contain *Prunus*, which all were grouped within subfamily Amygdaloideae of morphological taxonomy. In addition, *Fragaria* (Potentilleae) and *Rosa* (Roseae) as sister, which revealed have a close relationship within subfamily Rosoideae. Among these relationships of genera are consistent with previous research [62,63,64]. Amygdaloideae and Rosoideae are two large subfamilies in Rosaceae, which including more than 1000 and 2000 species [65], respectively. Until recently, a lot of research has been focus on molecular phylogenetic studies in Rosaceae, to provide a theoretical basis of phylogenetic relationships [66]. However, Rosaceae includes about 100 genera and 3000 species [67], the relationships among them are still obscure, which makes phylogenetic analysis with difficulty. In this study, *M. hupehensis* is one of *Malus* species, phylogenetic tree showed that the chloroplast genome of it clustered most closely with *M. baccata*, *M. micromalus*, and *M. prunifolia* than with *M. tschonoskii* in Figure 6. The result here roughly agrees with previous studies [22] and, besides, this conclusion from in terms of genomics. Until now, little has been known about the chloroplast genome of the *Malus*, and a limited number of chloroplast genome sequences of the *Malus* species are recorded in GeneBank, which poses limitations for studying the phylogenetic relationships within the genus. Overall, *M. hupehensis* cp genome sequences are useful for genomic information studies, enhancing the understanding of the phylogenetic relationships of the *Malus* species.

## 3. Materials and Methods

### 3.1. Plant Materials and DNA Sequencing

Fresh leaves of a single individual of *Malus hupehensis* were collected from Yangling (34°30′49′′ N, 108°04′06′′ E), Shaanxi Province, China. A voucher specimen (AF-06-19) of *M. hupehensis* has been deposited in the Institute of College of Horticulture, Northwest A and F University, Yangling, China. The leaves were immediately preserved in liquid nitrogen before DNA extraction. The total genomic DNA was isolated with the DNeasy Plant Mini Kit (Qiagen, Valencia, CA, USA), following the manufacturer’s instructions. Subsequently, the concentration and quality of the extracted DNA were checked and inspected using spectrophotometry and agarose gel electrophoresis, respectively. Genome sequencing was carried out on the Illumina Hiseq X Ten platform, following the manufacturer’s protocol (Illumina, San Diego, CA, USA). Approximately 24,794,523 clean reads were obtained, with a quality value ≥Q30, accounting for 95.10%.

### 3.2. Genome Assembly and Genome Annotation

Before chloroplast genome assembly, adapters and low-quality sequences were removed. The MITObim v1.8 program (https://github.com/chrishah/MITObim) was used to genome assembly, based on the remaining clean data [68], and the reference sequence from the *Malus baccata* cp genome (Genebank accession number: KX499859). The complete *Malus hupehensis* chloroplast genome sequence was annotated using the online software, Dual Organellar GenoMe Annotator (DOGMA, http://dogma.ccbb.utexas.edu/) [35], and then manually corrected by comparing it with the complete cp genomes of the other published *Malus* species in Geneious R 11.0.4 (Biomatters Ltd., Auckland, New Zealand) [69]. Finally, the circular chloroplast genome map was completed using the online program, OGDRAW (http://ogdraw.mpimp-golm.mpg.de/) [70].

### 3.3. Sequence Analysis

Codon usage was determined for all protein-coding genes. To examine the deviations in the synonymous codon usage, the relative synonymous codon usage (RSCU) and GC content were determined using MEGA 6 software (Department of Biological Sciences, Tokyo, Japan) [71]. We used the online REPuter [72] software (University of Bielefeld, Bielefeld, Germany) to identify repeats (forward, palindrome, complement and reverse sequences). The minimal repeat size was set as 30 bp, and the identity of repeats was greater than 90% (hamming distance equal to 3). Perl script MISA (http://pgrc.ipk-gatersleben.de/misa/misa.html) [73] was used to detect microsatellites with minimal repeat numbers of 10, 5, 4, 3, 3, and 3 for mononucleotide, dinucleotide, trinucleotide, tetranucleotide, pentanucleotide, and hexanucleotide repeats, respectively.

### 3.4. Comparative Genome Analysis 

The chloroplast genome size and organization were compared, and the differences of the IR border of nine *Malus* chloroplast genomes were analyzed. The *M. hupehensis* cp genome was used as a reference and was compared with other eight *Malus* species cp genomes using mVISTA software (Stanford University, Stanford, CA, USA) [74]. The whole-genome alignment for the cp genomes of eight species in the *Malus* genus, including *M. hupehensis* (MK020147), *M. trilobata* (KX499858), *M. florentina* (KX499862), *M. tschonoskii* (KX499863), *M. baccata* (KX499859), *M. micromalus* (MF062434), *M. prunifolia* (KU851961), *M. doumeri* (KX499861), and *M. yunnanensis* (MH394388) were analyzed.

### 3.5. Phylogenetic Analysis

The complete cp genome sequences of 26 species were downloaded from GenBank, using all genomes to ascertain the phylogenetic position of *Malus hupehensis*. Sequences were aligned using the MAFFT algorithm on the MAFFT version 7 alignment server (Osaka University, Suita, Japan) [75]. The maximum likelihood (ML) phylogenetic tree was generated using the MEGA 6 program (Department of Biological Sciences, Tokyo, Japan) [71], of which the bootstrap values of 1000 replicates to assess the branch support. In addition, *Ficus racemosa* and *Morus mongolica* (Moraceae) were set to the outgroup.

## 4. Conclusions

*M. hupehensis* is an economically important crabapple of the *Malus* genus in the Rosaceae family. In this study, we sequenced and annotated the whole chloroplast genome of *Malus hupehensis*, detected the arrangement of the genes, identified the SSRs and long repeats, and compared eight other complete chloroplast genomic characteristics of the *Malus* genus. *M. hupehensis* chloroplast genomes exhibited a typical quadripartite and circular structure in *Malus*, which is similar to those in other *Malus* species. The phylogenetic ML tree indicated that *Malus* was closely related to *Pyrus*, followed by *Prunus*, which indicated our data supports the position of *Malus* in the Amygdaloideae. Plus, the close relationships between *Fragaria* and *Rosa* were clustered into the clade as sister. The phylogenetic status of these genus is consistent with the previous report [48]. Because of the variety of *Malus* germplasm, the identification of evolutionary relationships is still vague, which has attracted a growing number of researchers that are trying to use biological, morphological, and molecular genetic classification analysis to classify *Malus* germplasm [21,76,77,78,79]. In this paper, *M. hupehensis* has a close relationship with *M. baccata*, *M. micromalus* and *M. prunifolia* than with *M. tschonoskii*. As recorded in book of Flora of China, *M. hupehensis* is similar to *M. baccata*. However, the leaf blade, calyx, and peduncle are slight purplish red, and the leaf edge is more acute, which are main distinguishing factors in two species. In the past, the AFLP marker system was used to analyze the genetic diversity of *Malus*, which indicated *M. hupehensis* and *M. baccata* within a group [80]. The *matK* sequence cluster analysis result indicated that *M. hupehensis*, *M. baccata*, and *M. micromalus* have a close relationship, *M. doumeri* and *M. yunnanensis* are within one clade, *M. trilobata* is closely related to *M. florentina*, and its sequence data also suggested *M. hupehensis* was close *M. baccata* [22]. Furthermore, our results are identical with the SRAP analysis, which indicated that *M. hupehensis*, *M. doumeri*, and *M. yunnanensis* are in different cluster groups [81]. China is an important primary area with rich *Malus* germplasm resources, with 17 wild species [82], including *M. hupehensis*, *M. baccata*, *M. manshurica*, *M. kansuensis*, *M. rockii*, *M. sikkimensis*, *M. sieboldii*, *M. transitoria*, *M. sieverii*, *M. komarovii*, *M. melliana*, *M. xiaojinensis*, *M. toringoides*, *M. yunnanensis*, *M. ombrophila*, *M. honanensis*, and *M. prattii*. It is necessary for more research of the complete cp genome within the *Malus* genus in the future. Obtaining the chloroplast genome of *Malus hupehensis*, which provided a possibility for further study to compare all wild *Malus* species in China, and other *Malus* species. In addition, our data also can provide a useful molecular basis, which can facilitate more extensive contributions to the exploration of the variation of *Malus* populations and further more studies.

## Figures and Tables

**Table 1:** Summary of complete chloroplast genomes for nine *Malus* species.

Genome Characteristics	*M. hupehensis*	*M. trilobata*	*M. florentina*	*M. tschonoskii*	*M. baccata*	*M. micromalus*	*M. prunifolia*	*M. doumeri*	*M. yunnanensis*
Accession number	MK020147	KX499858	KX499862	KX499863	KX499859	MF062434	KU851961	KX499861	MH394388
Genome size (bp)	160,065	160,207	159,712	160,053	160,163	159,834	160,041	159,584	160,068
LSC length (bp)	88,166	88,107	87,710	88,137	88,267	87,950	88,119	87,670	88,245
SSC length (bp)	19,193	19,316	19,250	19,210	19,188	19,176	19,204	19,168	19,211
IR length (bp)	26,353	26,392	26,376	26,353	26,354	26,354	26,359	26,373	26,306
No. of different genes	112	110	110	110	109	111	111	110	112
No. of different protein-coding genes	78	76	77	77	76	77	77	77	78
No. of different tRNA genes	30	30	29	29	29	30	30	29	30
No. of different rRNA genes	4	4	4	4	4	4	4	4	4
% GC content in LSC	34.2	34.2	34.3	34.2	34.2	34.3	34.2	34.4	34.2
% GC content in SSC	30.4	30.3	30.4	30.4	30.4	30.4	30.4	30.4	30.4
% GC content in IR	42.7	42.6	42.6	42.7	42.7	42.7	42.7	42.6	42.7
% GC content of genome	36.6	36.5	36.6	36.5	36.5	36.6	36.6	36.6	36.5

**Table 2:** Gene contents of the *M. hupehensis* chloroplast genome, based on genome annotation.

Group of Genes	Gene Name
DNA-dependent RNA polymerase	*rpoA*, *rpoB*, *rpoC1*^#^, *rpoC2*
tRNA genes	*trnA-UGC*^#^ (×2), *trnC-GCA*, *trnD-GUC*, *trnE-UUC*, *trnF-GAA*, *trnG-GCC ^#^*, *trnG-UCC*, *trnH-GUG*, *trnI-CAU* (×2), *trnI-GAU* ^#^ (×2), *trnK-UUU* ^#^, *trnL-CAA* (×2), *trnL-UAA* ^#^, *trnL-UAG*, *trnfM-CAU*, *trnM-CAU*, *trnN-GUU* (×2), *trnP-GGG*, *trnP-UGG*, *trnQ-UUG*, *trnR-ACG* (×2), *trnR-UCU*, *trnS-GCU*, *trnS-GGA*, *trnS-UGA*, *trnT-GGU*, *trnT-UGU*, *trnV-GAC* (×2), *trnV-UAC* ^#^, *trnW-CCA*, *trnY-GUA*
Ribosomal small subunit	*rps2*, *rps3*, *rps4*, *rps7* (×2), *rps8*, *rps11*, *rps12* ^#^ (×2), *rps14*, *rps15*, *rps16* ^#^, *rps18*, *rps19* (×2)
Ribosomal large subunit	*rpl2*^#^ (×2), *rpl14*, *rpl16 ^#^*, *rpl20*, *rpl22*, *rpl23* (×2), *rpl32*, *rpl33*, *rpl36*
rRNA genes	*rrn16* (×2), *rrn23* (×2), *rrn4.5* (×2), *rrn5* (×2)
ATP synthase	*atpA*, *atpB*, *atpE*, *atpF*^#^, *atpH*, *atpI*
Photosystem I	*psaA*, *psaB*, *psaC*, *psaI*, *psaJ*
Photosystem II	*psbA*, *psbB*, *psbC*, *psbD*, *psbE*, *psbF*, *psbH*, *psbI*, *psbJ*, *psbK*, *psbL*, *psbM*, *psbN*, *psbT*, *psbZ*
NADH dehydrogenase	*ndhA*^#^, *ndhB*^#^ (×2), *ndhC*, *ndhD*, *ndhE*, *ndhF*, *ndhG*, *ndhH*, *ndhI*, *ndhJ*, *ndhK*
Cytochrome b/f complex	*petA*, *petB*^#^, *petD ^#^*, *petG*, *petL*, *petN*
Large subunit of rubisco	*rbcL*
Maturase	*matK*
Subunit of acetyl-CoA carboxylase	*accD*
Envelope membrane protein	*cemA*
Protease	*clpP* ^##^
c-type cytochrome synthesis	*ccsA*
Conserved open reading frames	*ycf1* (×2), *ycf2* (×2), *ycf3* ^##^, *ycf4*

^#^ genes with one intron, ^##^ genes with two introns, Genes in the IR regions are followed by the (×2) symbol.

**Table 3:** Location and length of intron-containing genes within the *M. hupehensis* chloroplast genome.

Gene	Location	ExonI (bp)	IntronI (bp)	ExonII (bp)	IntronII (bp)	ExonIII (bp)
*trnK-UUU*	LSC	37	2497	35		
*trnG-UCC*	LSC	23	698	48		
*trnL-UAA*	LSC	37	514	50		
*trnV-UAC*	LSC	39	592	37		
*trnI-GAU*	IR	42	943	35		
*trnA-UGC*	IR	38	807	35		
rps12 *	LSC	114	-	232	541	26
*rps16*	LSC	40	864	221		
*rpl16*	LSC	9	983	399		
*rpl2*	IR	390	686	435		
*rpoC1*	LSC	435	741	1611		
*ndhA*	SSC	552	1134	540		
*ndhB*	IR	777	669	756		
*ycf3*	SSC	126	708	228	744	153
*petB*	LSC	6	797	642		
*atpF*	LSC	144	737	411		
*clpP*	LSC	71	826	292	627	228
*petD*	LSC	8	724	475		

Note. *rps12* * gene is a trans-spliced gene with the two duplicated 3′ end exons in the IR regions and a 5′ end exon in the LSC region.

## Supplementary Materials

The following are available online. Table S1, Codon–anticodon recognition pattern and codon usage in the *M. hupehensis* chloroplast genome; Table S2, Long repeat sequences in the *M. hupehensis* chloroplast genome.

## Abbreviations

cp	Chloroplast
PCGs	Protein-coding genes
tRNAs	Transfer RNA genes
rRNAs	Ribosomal RNA genes
IRs	Inverted repeat regions
LSC	Large single copy
SSC	Small single copy
SSR	Simple sequence repeat
ML	Maximum likelihood
