# Supplementary material for: Complete Chloroplast Genome Sequence of Malus hupehensis: Genome Structure, Comparative Analysis, and Phylogenetic Relationships
Source: Molecules. 2018 Nov 8;23(11):2917. doi: 10.3390/molecules23112917 (PMC6278565; doi:10.3390/molecules23112917)
Supplement: Supplementary file 1 [file molecules-23-02917-s001.pdf]

molecules

MDPI

Table S1. Codon–anticodon recognition pattern and codon usage in the *M. hupehensis* chloroplast genome.

Amino Acid	Codon	No.	RSCU*	tRNA	Amino Acid	Codon	No.	RSCU*	tRNA
Phe	UUU	963	1.3		Tyr	UAU	786	1.61	
Phe	UUC	516	0.7	*trnF-GAA*	Tyr	UAC	190	0.39	*trnY-GUA*
Leu	UUA	889	1.94	*trnL-UAA*	Stop	UAA	48	1.71	
Leu	UUG	563	1.23	*trnL-CAA*	Stop	UAG	21	0.75	
Leu	CUU	583	1.27		His	CAU	486	1.55	
Leu	CUC	179	0.39		His	CAC	141	0.45	*trnH-GUG*
Leu	CUA	357	0.78	*trnL-UAG*	Gln	CAA	717	1.54	*trnQ-UUG*
Leu	CUG	176	0.38		Gln	CAG	215	0.46	
Ile	AUU	1103	1.47		Asn	AAU	976	1.53	
Ile	AUC	434	0.58	*trnI-GAU*	Asn	AAC	297	0.47	*trnN-GUU*
Ile	AUA	717	0.95	*trnI-CAU*	Lys	AAA	1043	1.49	*trnK-UUU*
Met	AUG	617	1	*trn(f)M-CAU*	Lys	AAG	355	0.51	
Val	GUU	521	1.46		Asp	GAU	880	1.62	
Val	GUC	159	0.45	*trnV-GAC*	Asp	GAC	207	0.38	*trnD-GUC*
Val	GUA	547	1.53	*trnV-UAC*	Glu	GAA	1008	1.48	*trnE-UUC*
Val	GUG	200	0.56		Glu	GAG	355	0.52	
Ser	UCU	565	1.69		Cys	UGU	225	1.5	
Ser	UCC	323	0.97	*trnS-GGA*	Cys	UGC	75	0.5	*trnC-GCA*
Ser	UCA	401	1.2	*trnS-UGA*	Stop	UGA	15	0.54	
Ser	UCG	185	0.55		Trp	UGG	450	1	*trnW-CCA*
Pro	CCU	413	1.54		Arg	CGU	338	1.28	*trnR-ACG*
Pro	CCC	200	0.75		Arg	CGC	113	0.43	
Pro	CCA	308	1.15	*trnP-UGG*	Arg	CGA	365	1.38	
Pro	CCG	149	0.56		Arg	CGG	121	0.46	
Thr	ACU	540	1.61		Arg	AGA	406	1.21	*trnR-UCU*
Thr	ACC	244	0.73	*trnT-GGU*	Arg	AGG	127	0.38	
Thr	ACA	412	1.23	*trnT-UGU*	Ser	AGU	483	1.82	
Thr	ACG	148	0.44		Ser	AGC	170	0.64	*trnS-GCU*
Ala	GCU	643	1.84		Gly	GGU	580	1.31	
Ala	GCC	216	0.62		Gly	GGC	183	0.41	*trnG-GCC*
Ala	GCA	387	1.11	*trnA-UGC*	Gly	GGA	715	1.62	*trnG-UCC*
Ala	GCG	149	0.43		Gly	GGG	290	0.66	

Note. RSCU: Relative Synonymous Codon Usage. * Numerals indicate the frequency of usage of each codon in 26,188 codons of 84 potential protein-coding genes.

Table S2. Long repeat sequences in the *M. hupehensis* chloroplast genome.

ID	Size (bp)	Repeat Start 1	Type	Repeat Start 2	E-value	Gene	Region
1	30	8387	P	48,214	6.25×10^{-9}	IGS; *trnS-GGA*	LSC
2	30	10,416	F	10,436	5.63×10^{-7}	IGS	LSC
3	30	7031	P	75,053	2.45×10^{-5}	IGS; *clpP* (intron)	LSC
4	30	10,493	F	10,510	2.45×10^{-5}	IGS	LSC
5	30	10,640	R	10,640	2.45×10^{-5}	IGS	LSC
6	30	114,054	P	114,054	2.45×10^{-5}	ycf1	IRB
7	30	114,054	F	134,147	2.45×10^{-5}	ycf1	IRB; IRA
8	30	134,147	P	134,147	2.45×10^{-5}	ycf1	IRB
9	30	7031	P	15,592	6.85×10^{-4}	IGS	LSC
10	30	7035	F	81,799	6.85×10^{-4}	IGS	LSC
11	30	9925	F	9,946	6.85×10^{-4}	IGS	LSC
12	31	0	F	37	6.54×10^{-6}	IGS	LSC
13	31	7028	R	81,797	1.90×10^{-4}	IGS	LSC
14	31	8386	F	38,434	1.90×10^{-4}	IGS	LSC
15	31	10,542	F	10,582	1.90×10^{-4}	IGS	LSC
16	31	34,527	F	34,550	1.90×10^{-4}	IGS	LSC
17	31	50,525	P	50,525	1.90×10^{-4}	IGS	LSC
18	31	62,305	F	62,330	1.90×10^{-4}	IGS	LSC
19	32	50,142	P	50,142	3.91×10^{-10}	IGS	LSC
20	32	86,654	F	86,686	3.91×10^{-10}	IGS	LSC
21	32	34,540	P	34,550	5.23×10^{-5}	IGS	LSC
22	32	41,767	F	43,991	5.23×10^{-5}	*PsaB; psaA*	LSC
23	32	54,786	F	54,825	5.23×10^{-5}	IGS	LSC
24	32	93,104	F	93,125	5.23×10^{-5}	ycf2	IRB
25	32	93,104	P	155,074	5.23×10^{-5}	ycf2	IRB; IRA
26	32	93,125	P	155,095	5.23×10^{-5}	ycf2	IRB; IRA
27	32	155,074	F	155,095	5.23×10^{-5}	ycf2	IRA
28	33	111,797	F	111,828	4.64×10^{-7}	IGS	IRB
29	33	111,797	P	136,370	4.64×10^{-7}	IGS	IRB; IRA
30	33	111,828	P	136,401	4.64×10^{-7}	IGS	IRB; IRA
31	33	136,370	F	136,401	4.64×10^{-7}	IGS	IRA
32	33	14,023	P	14,023	1.44×10^{-5}	IGS	LSC
33	34	54,636	F	54,670	2.49×10^{-9}	IGS	LSC
34	34	95,526	F	95,544	1.23×10^{-7}	ycf2	IRB
35	34	95,526	P	152,653	1.23×10^{-7}	ycf2	IRB; IRA
36	34	95,544	P	152,671	1.23×10^{-7}	ycf2	IRB; IRA
37	34	152,653	F	152,671	1.23×10^{-7}	ycf2	IRA
38	36	10,836	R	10,846	2.94×10^{-7}	IGS	LSC
39	38	39,274	F	39,301	9.54×10^{-14}	IGS	LSC
40	38	46,752	F	125,524	1.09×10^{-11}	*ycf3* (intron); *ndhA* (intron)	LSC; IRA
41	39	46,752	F	102,849	2.79×10^{-12}	*ycf3* (intron); IGS	LSC; IRB
42	39	46,752	P	145,343	2.79×10^{-12}	*ycf3* (intron); IGS	LSC; IRA
43	40	102,847	F	125,522	5.96×10^{-15}	IGS; *ndhA* (intron)	IRB; IRA
44	40	125,522	P	145,344	5.96×10^{-15}	*ndhA* (intron); IGS	SSC; IRA
45	44	78,131	P	78,131	2.33×10^{-17}	IGS	LSC
46	45	225	P	225	7.86×10^{-16}	IGS	LSC
47	45	54,842	R	54,842	5.19×10^{-14}	IGS	LSC
48	48	10,290	P	10,290	9.23×10^{-16}	IGS	LSC
49	63	56,004	P	56,004	1.60×10^{-26}	IGS	LSC

F: forward repeat; P: palindrome repeat; R: reverse IGS: intergenic space.
